# Area-level social determinants of health and individual-level social risks: Assessing predictive ability and biases in social risk screening

**DOI:** 10.1017/cts.2023.680

**Published:** 2023-11-10

**Authors:** Wyatt P. Bensken, Brenda M. McGrath, Rachel Gold, Erika K. Cottrell

**Affiliations:** 1 Department of Research, OCHIN, Portland, OR, USA; 2 Quantitative Sciences Core, OCHIN, Portland, OR, USA; 3 Kaiser Permanente Center for Health Research, Portland, OR, USA; 4 Oregon Health and Science University, Portland, OR, USA

**Keywords:** Social determinants of health, social risks, area deprivation, health inequities, community health centers

## Abstract

**Introduction:**

Area-level social determinants of health (SDoH) and individual-level social risks are different, yet area-level measures are frequently used as proxies for individual-level social risks. This study assessed whether demographic factors were associated with patients being screened for individual-level social risks, the percentage who screened positive for social risks, and the association between SDoH and patient-reported social risks in a nationwide network of community-based health centers.

**Methods:**

Electronic health record data from 1,330,201 patients with health center visits in 2021 were analyzed using multilevel logistic regression. Associations between patient characteristics, screening receipt, and screening positive for social risks (e.g., food insecurity, housing instability, transportation insecurity) were assessed. The predictive ability of three commonly used SDoH measures (Area Deprivation Index, Social Deprivation Index, Material Community Deprivation Index) in identifying individual-level social risks was also evaluated.

**Results:**

Of 244,155 (18%) patients screened for social risks, 61,414 (25.2%) screened positive. Sex, race/ethnicity, language preference, and payer were associated with both social risk screening and positivity. Significant health system-level variation in both screening and positivity was observed, with an intraclass correlation coefficient of 0.55 for social risk screening and 0.38 for positivity. The three area-level SDoH measures had low accuracy, sensitivity, and area under the curve when used to predict individual social needs.

**Conclusion:**

Area-level SDoH measures may provide valuable information about the communities where patients live. However, policymakers, healthcare administrators, and researchers should exercise caution when using area-level adverse SDoH measures to identify individual-level social risks.

## Introduction

Social determinants of health (SDoH), defined as the conditions in which people “are born, grow, live, work, and age [1],” include factors such as neighborhood conditions, access to housing, food, and transportation and have a profound impact on health outcomes [2–4]. These area-level SDoH are shaped by underlying structural factors and can have both positive and negative impacts on health. When these area-level SDoH become adverse, they may present as specific individual-level adverse social conditions that have a negative impact on health (e.g., unstable housing) which are termed *social risks* [5]. Healthcare providers are increasingly incorporating knowledge of these social risk factors into clinical care, [6–8] and policymakers are considering including such factors in risk adjustment, value-based purchasing policies, and quality measures [9–11]. For example, there are two recently released quality measures available for a few payment programs that are specifically focused on social risk screening and positivity [12,13]. However, there remain important knowledge gaps about the relationship between area-level adverse SDoH and individual-level social risks which may be perceived to represent similar concepts but in fact are different [14–18].

Area-level measures of SDoH, such as the Area Deprivation Index (ADI), Social Deprivation Index (SDI), and the Material Community Deprivation Index (MCDI) among others, [19] are readily and publicly available. These measures are assessed at different geographical levels (e.g., Census tract, ZIP code tabulation area, county) and can be linked to individual-level clinical data using information on patient addresses. Area-level measures are often used to target policy initiatives and community-level investment and are increasingly being considered as a way to account for social risk in healthcare payment.

In recent years, there have been efforts to incorporate social risk screening into clinical care and document results in electronic health records; yet the collection and documentation of social risk screening data is far from universal due to several barriers [20–23]. Thus, individual-level social risk measures remain under-collected in healthcare settings, limiting their use in research and policy. This all has resulted in considerable heterogeneity in the way social risk is measured and a need for additional research on the relationship between area-level measures and individual-level social risks. The limited research to date found poor congruence between one *area-level* measure and *individual-level* social risks [24]. It is unclear how these relationships vary when using *different* area-level measures [25]. Exploring this relationship can help elucidate the relationship between area- and individual-level measures of the social drivers of health.

To that end, these analyses examined the variation of social risk screening practices across a nationwide network of community-based health centers, then quantified the relationship between area-level SDoH and individual-level social risks. Results are intended to provide evidence regarding the relationship between area-level SDoH and individual-level social risks for health care, research, and policy purposes. We focus on community healthcare settings which annually serve>30 million low-income and underserved patients across the United States [26], a population that disproportionately experiences area-level adverse SDoH and social risks [27].

## Methods

Study data were obtained from OCHIN, Inc., a nationwide network of community-based healthcare organizations that share a single instance of the Epic© electronic health record (EHR). OCHIN member health centers primarily serve minoritized patient populations, and their data capture a population that has historically been missing in research (e.g., uninsured persons). This study included adult (≥ 18 years of age) patients with an in-person or telehealth ambulatory visit in 2021 who also had a geocoded address that could be linked to area-level SDoH data. An individual’s latest visit in 2021 was their index date. EHR data provided patient’s sex, date of birth, race / ethnicity, primary language, federal poverty level (FPL) percentage, primary payor at index visit, patient-reported social risk screening results (see below), and patient address at index visit, which was geocoded to Census tract.

### Area-Level Measure of SDoH

Based on recent work on commonly used area-level SDoH indices [19], we selected the three that were readily publicly available and could be calculated at the Census tract level: the Area Deprivation Index (ADI) [28], Social Deprivation Index (SDI) [29], and Material Community Deprivation Index (MCDI) [30]. The ADI is a composite score of 17 Census indicators weighted by a factor score, and when using the *sociome* R package [31] ranges from 38.5 to 211.8 for all Census tracts using the 2019 American Community Survey (ACS). The calculated ADI for each Census tract used the state of residence as the reference level. This means all ADI values are relative to the rest of the state to ensure a measure of deprivation that is more appropriate for a localized context [31,32]. The SDI is a composite of seven indicators from the ACS and ranges from 0 to 100. The MCDI is a composite measure that includes five demographic characteristics from the ACS and ranges from 0 to 1. For all three of the included measures, a higher score represents a more deprived area. Table 1 presents the indicators included in each of the SDoH indices.

**Table 1.** Area-level SDoH domains and census variables in each measure

	ADI	SDI	MCDI
**Income**			
% of families below FPL	X	X	X
Median family income	X		X
Income disparity	X		
% of population below 150% FPL	X		
% of population below 150% FPL			X
**Employment**			
% of unemployed adults below the age of 65	X	X	
% employed persons ≥16 years in white-collar occupations	X		
**Education**			
% adults ≥25 years with less than 9 years of education	X		
% adults ≥25 years with at least a high school diploma	X		X
% with less than 12 years of education		X	
**Household Structure**			
% of single-parent households	X	X	
% living in overcrowded housing units	X	X	
% households without a telephone	X		
% of houses that are vacant			X
**Housing and Environment**			
Median home value	X		
Median gross rent	X		
Median monthly mortgage	X		
% owner-occupied housing units	X		
% occupied housing units without complete plumbing	X		
% of people residing in rented housing units		X	
**Transportation**			
% of households without a car	X	X	
**Insurance**			
% with no health insurance coverage			X

ADI = Area Deprivation Index; MCDI = Material Community Deprivation Index; SDI = Social Deprivation Index.

### Individual-Level Social Risk Screening

In the OCHIN network, health centers can choose from several [33,34] social risk screening tools or develop their own. All included social risk questions have been categorized into domains including child/family care, education, employment, financial strain, food insecurity, health literacy, housing instability, housing quality, relationship safety, social isolation, transportation insecurity, and utilities insecurity. There is meaningful variation in social risk screening practices across health centers [20,35]. For example, health centers conduct screening at different workflow steps (i.e., prior to visit, upon check-in, while rooming), and patients may complete screening forms themselves or be screened by rooming staff or other care team members. Further, health centers use different tools or may ask their own screening questions, yielding varying responses that indicate a “positive” screening result (indicating the presence of a social risk). However, the variation in questions/tools has been mapped to a harmonized set of social risk domains and to a positive/negative response in the EHR.

This paper describes both patients who were *screened* and those who *screened positive*, indicating that the patient reported the presence of a social risk. These were defined, respectively, as whether a social risk screening (in any domain) occurred within 12 months of the patient’s latest encounter in 2021 (Supplemental Figure 1), and among those screened, whether a patient screened positive for any social risk domain within this time frame.

### Statistical Analyses

We first describe the study population, highlighting differences in those who were screened and, among those screened, who screened positive for social risks. Next, multilevel logistic regression analysis examined demographic factors associated with being screened and with screening positive for social risks. As race/ethnicity and language were highly correlated (Supplemental Table 1), we analyzed these covariates in separate models, in which patients were nested within health systems. In this network, health systems are the organizational unit that may have multiple physical locations (health centers or clinics). We nested within the health system recognizing that it is often organization-wide practices (e.g., intake procedures) that drive this and to ensure model convergence. Additionally, we examined health system-level variation in social risk screening by assessing the intraclass correlation (ICC), i.e., degree of variation in the outcome that can be attributed to the health system. We next calculated Pearson correlation coefficients of the strength and direction of the associations between these three indices. Finally, we describe variation in the three SDoH measures across different demographic groups to assess how these measures vary across these groups.

To evaluate the predictive capability of these area-level SDoH measures in identifying individuals with social risks, we used a simple random sampling approach to split the 244,155 screened patients into a 70% (*n* = 170,908) training sample and 30% (*n* = 73,247) test sample. A comparison of the training and test samples is shown in Supplemental Table 2, which demonstrates that the training and test samples were balanced on all covariates and outcomes included in the analyses. Using Youden’s Index [36] on the training data, we determined the optimal value of the area-level SDoH measure for differentiating between individuals with and without social risks. We then applied this value to predict social risks in the test data and reported the accuracy, sensitivity, and specificity of the prediction. All analyses were conducted with a 95% confidence interval.

Data cleaning procedures were performed using SQL Server Management Studio and SAS version 9.4, while R version 4.1.1 was used for data analysis. This study was approved by the Advarra Institutional Review Board.

## Results

### Study Population

The study dataset included 1,395,345 adult patients who had an in-person or telehealth ambulatory visit in 2021. Of these, 95% had geocoded address data, and of these 1,330,201 had a valid ADI, SDI, or MCDI. Study data came from 113 health systems across 22 states (Alaska, California, Colorado, Connecticut, Georgia, Idaho, Illinois, Indiana, Louisiana, Massachusetts, Minnesota, Missouri, Montana, North Carolina, New Jersey, New York, Ohio, Oregon, South Carolina, Texas, Washington, and Wisconsin).

In this study population, 16.6% of patients were Black/African American, and 34.1% were Hispanic/Latino (Table 2). Similarly, while English was the predominant preferred language, 22.9% of patients preferred care in Spanish (Table 2). A substantial proportion (42.4%) of the patients were younger than 40. Overall, 52.2% of patients were at or below the FPL, 20.5% of patients were uninsured and 43.8% were insured through Medicaid (Table 2).

**Table 2.** Description of study population, social risk screening, and social risk positivity

	Study population	Social risk screening		Social risk screening, positive	
		**No**	**Yes**	**No**	**Yes**
	*n* = 1,330,201	*n* = 1,086,046	*n* = 244,155	*n* = 182,741	*n* = 61,414
**Sex, n (%)**					
Female	779814 (58.6)	617038 (56.8)	162776 (66.7)	126639 (69.3)	36,137 (58.8)
Male	549346 (41.3)	468142 (43.1)	81,204 (33.3)	56,011 (30.7)	25,193 (41.0)
Other/Missing/Unknown	1041 (0.1)	866 (0.1)	175 (0.1)	91 (0.0)	84 (0.1)
**Race/Ethnicity, n (%)**					
AIAN	7018 (0.5)	5866 (0.5)	1152 (0.5)	691 (0.4)	461 (0.8)
Asian	94,853 (7.1)	84,438 (7.8)	10,415 (4.3)	8061 (4.4)	2354 (3.8)
Black/AA	220591 (16.6)	172749 (15.9)	47,842 (19.6)	32,637 (17.9)	15,205 (24.8)
Hispanic/Latino	452948 (34.1)	363391 (33.5)	89,557 (36.7)	73,585 (40.3)	15,972 (26.0)
Multiple races	11,054 (0.8)	9291 (0.9)	1763 (0.7)	1060 (0.6)	703 (1.1)
NHOPI	4687 (0.4)	4037 (0.4)	650 (0.3)	492 (0.3)	158 (0.3)
Other/Missing/Unknown	91,030 (6.8)	79,067 (7.3)	11,963 (4.9)	8837 (4.8)	3126 (5.1)
White	448020 (33.7)	367207 (33.8)	80,813 (33.1)	57,378 (31.4)	23,435 (38.2)
**Age, n (%)**					
18–29	294737 (22.2)	243019 (22.4)	51,718 (21.2)	40,154 (22.0)	11,564 (18.8)
30–39	269033 (20.2)	218415 (20.1)	50,618 (20.7)	38,300 (21.0)	12,318 (20.1)
40–49	236686 (17.8)	187026 (17.2)	49,660 (20.3)	37,734 (20.6)	11,926 (19.4)
50–64	337582 (25.4)	278481 (25.6)	59,101 (24.2)	40,304 (22.1)	18,797 (30.6)
65+	192163 (14.4)	159105 (14.6)	33,058 (13.5)	26,249 (14.4)	6809 (11.1)
**Language, n (%)**					
English	922028 (69.3)	753018 (69.3)	169010 (69.2)	122783 (67.2)	46,227 (75.3)
Spanish	304612 (22.9)	244094 (22.5)	60,518 (24.8)	49,059 (26.8)	11,459 (18.7)
Other	91,900 (6.9)	78,588 (7.2)	13,312 (5.5)	9986 (5.5)	3326 (5.4)
Unknown/Missing	11,661 (0.9)	10,346 (1.0)	1315 (0.5)	913 (0.5)	402 (0.7)
**FPL Percentage**					
Mean (Std Dev)	99.6 (272.1)	97.7 (260.3)	108.2 (319.4)	113.5 (318.6)	93.1 (321.2)
Median [IQR]	56 [0, 116]	55 [0, 116]	60 [0, 118]	66 [0, 123]	38 [0, 105]
0	344755 (25.9)	285009 (26.2)	59,746 (24.5)	41,873 (22.9)	17,873 (29.1)
1–100	349182 (26.3)	282867 (26.0)	66,315 (27.2)	49,404 (27.0)	16,911 (27.5)
>100	302593 (22.7)	246243 (22.7)	56,350 (23.1)	43,968 (24.1)	12,382 (20.2)
Missing	333671 (25.1)	271927 (25.0)	61,744 (25.3)	47,496 (26.0)	14,248 (23.2)
**Payer, n (%)**					
Private Insurance	309189 (23.2)	250728 (23.1)	58,461 (23.9)	46,965 (25.7)	11,496 (18.7)
Medicare	165886 (12.5)	133472 (12.3)	32,414 (13.3)	24,856 (13.6)	7558 (12.3)
Medicaid	582331 (43.8)	473109 (43.6)	109222 (44.7)	79,146 (43.3)	30,076 (49.0)
Uninsured/Other	272795 (20.5)	228737 (21.1)	44,058 (18.0)	31,774 (17.4)	12,284 (20.0)

AIAN = American Indian or Alaska Native; AA = African American; FPL = federal poverty level; IQR = interquartile range; NHOPI = Native Hawaiian or Other Pacific Islander; Std Dev = standard deviation.

Of the 1,330,201 included patients, 244,155 (18.4%) were screened for social risks within 12 months of their index encounter. Of those who were screened, 61,414 (25.2%) screened positive for having a social risk (Table 2). The percentage of patients up to date on their social risk screening varied across health systems, ranging from 0% to 95%. The median screening rate was 25.1% (IQR: 2.3, 27.4).

### Demographics and Social Risk Screening

Both the distribution (Table 2) and adjusted regression model (Table 3) revealed variations in individual-level social risks across demographic groups. Males had lower adjusted odds of being screened (Table 3). Asian, Black/African American, Hispanic/Latino, and individuals of multiple races had higher odds of being screened than white individuals (Table 3). Those who preferred a language other than English had higher odds of being screened for social risks (Table 3). Adults aged 30–49 years had higher odds of being screened, and those aged 50+ years had lower odds, compared to those aged 18–29 years (Table 3). Individuals with Medicare or Medicaid had higher odds of being screened, and those who were uninsured had lower odds, compared to those with private insurance (Table 3). Finally, when compared to those who were above the FPL (> 100 FPL percentage), those who were below the FPL or did not have documented FPL data had lower odds of being screened (Table 3).

**Table 3.** Odds ratios (OR) and 95% confidence intervals for the association between demographics and being screened for social risks and having social needs

	Screened for social risks	Positive for social risk
	ICC = 0.55	ICC = 0.38
**Sex**		
Female	*Reference*	*Reference*
Male	0.59 (0.58, 0.59)	1.24 (1.21, 1.27)
Other/Missing	1.21 (1.04, 1.40)	1.91 (1.36, 2.67)
**Race/Ethnicity**		
White	*Reference*	*Reference*
AIAN	0.97 (0.90, 1.04)	1.30 (1.14, 1.50)
Asian	1.16 (1.13, 1.20)	0.55 (0.52, 0.59)
Black/AA	1.07 (1.05, 1.09)	1.05 (1.01, 1.08)
Hispanic/Latino	1.11 (1.09, 1.13)	0.80 (0.78, 0.83)
Multiple races	1.14 (1.07, 1.21)	1.40 (1.25, 1.57)
NHOPI	0.91 (0.83, 0.99)	0.73 (0.60, 0.90)
Other/Missing/Unknown	0.79 (0.77, 0.81)	1.02 (0.97, 1.08)
**Age**		
18–29	*Reference*	*Reference*
30–39	1.14 (1.12, 1.16)	1.08 (1.04, 1.12)
40–49	1.31 (1.29, 1.33)	1.17 (1.13, 1.21)
50–64	0.91 (0.90, 0.93)	1.49 (1.44, 1.54)
65+	0.87 (0.85, 0.89)	0.87 (0.83, 0.91)
**Payer**		
Private Insurance	*Reference*	*Reference*
Medicare	1.36 (1.33, 1.39)	1.50 (1.43, 1.57)
Medicaid	1.23 (1.21, 1.24)	1.89 (1.84, 1.95)
Uninsured/Other	0.83 (0.82, 0.85)	1.91 (1.84, 1.98)
**FPL Percentage**		
>100	*Reference*	*Reference*
0	0.85 (0.84, 0.86)	1.40 (1.35, 1.44)
1–100	0.97 (0.95, 0.98)	1.35 (1.31, 1.39)
Missing	0.75 (0.74, 0.76)	1.25 (1.21, 1.29)
**Language^1^**		
English	*Reference*	*Reference*
Spanish	1.12 (1.11, 1.14)	0.84 (0.82, 0.87)
Other	1.11 (1.09, 1.14)	0.61 (0.58, 0.64)
Unknown/No Information	0.64 (0.60, 0.68)	1.01 (0.87, 1.17)

AIAN = American Indian or Alaska Native; AA = African American; FPL = federal poverty level; ICC = intraclass correlation coefficient, which is the proportion of the variance in the outcome (screening or social risk positivity) driven by the health system; NHOPI = Native Hawaiian or Other Pacific Islander.

1 Due to the correlation between race/ethnicity and language, they were analyzed in two different sets of models. Each set of models contained sex, age, payer, and FPL percentage with the estimates displayed here from the model including race/ethnicity. The estimates for sex, age, payer, and FPL percentage from the model including language were nearly identical with the full model including language shown in Supplemental Table 3.

There were significant associations between sex, race/ethnicity, language, age, payer, and FPL and screening positive for social risks (Table 3). Males (compared to females), American Indian and Alaska Native (AIAN), and multiple race (compared to white), preferring care in English, age 30–39 years, 40–49 years, and 50–64 years (compared to 18–29 years), and having coverage by Medicare or Medicaid or being uninsured (compared to privately insured) were associated with higher odds of screening positive for social risks (Table 3). Conversely, Asian, Hispanic/Latino, and Native Hawaiian and Other Pacific Islander (NHOPI) race/ethnicity were associated with lower odds of screening positive for social risks compared to non-Hispanic/Latino white race/ethnicity. Older age (65+ years) was associated with lower odds of screening positive for social risks compared to age 18-29. Finally, those who were at or below the FPL had higher odds of screening positive for social risks, compared to those above the FPL. The ICC was 0.55 for the model evaluating screening, and 0.38 for the model evaluating positivity, suggesting that health system-level practices accounted for 55% and 38% of the variation in screening and positivity, respectively, *after* adjusting for patient demographics.

### Area-Level SDoH Measures

The three area-level SDoH measures were highly correlated with each other. The ADI and MCDI were the most correlated with a Pearson correlation coefficient of 0.93, followed by the ADI and SDI (0.87), and finally the SDI and MCDI (0.84).

**Figure 1.**
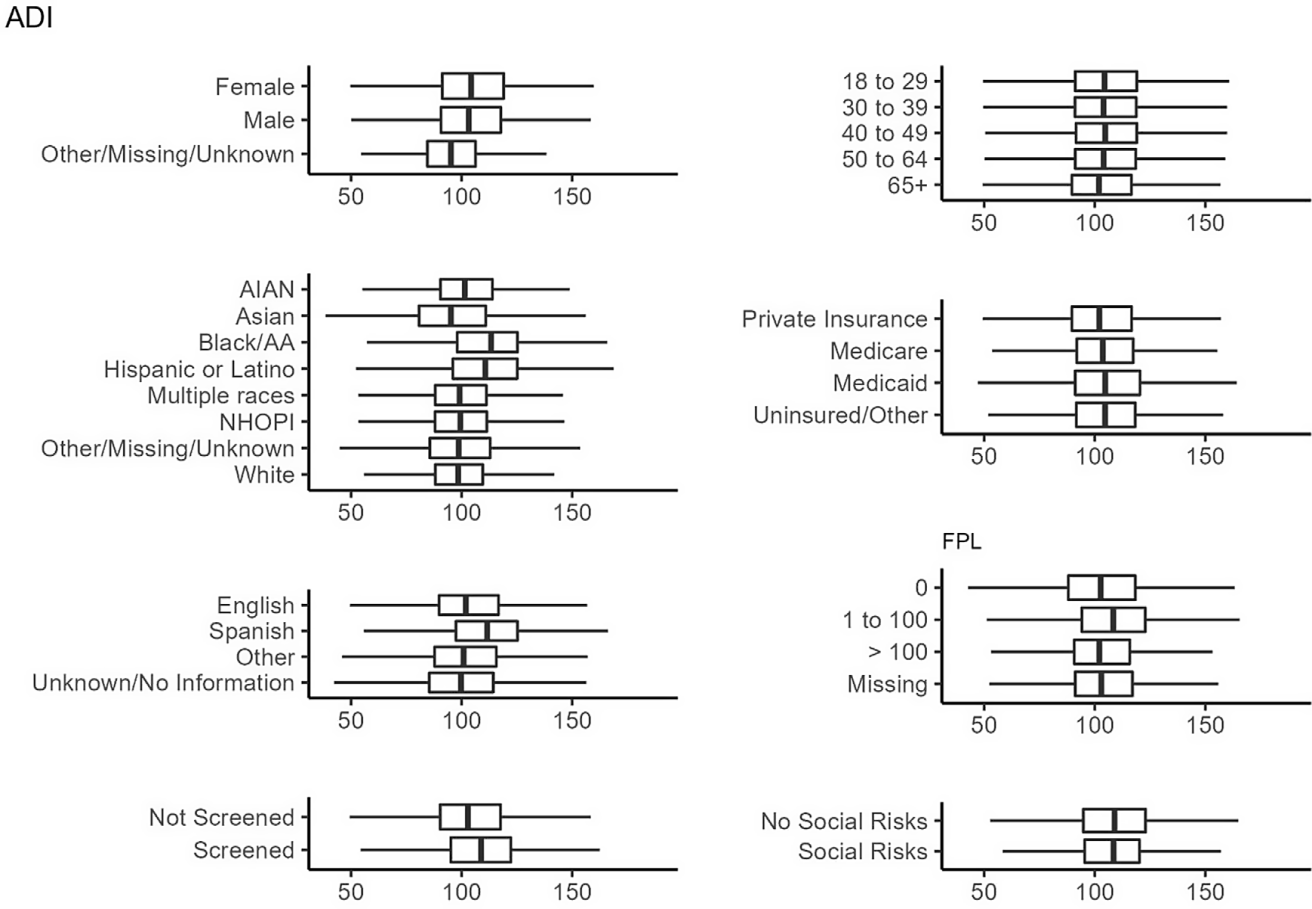
Distribution of the Area Deprivation Index (ADI) among the 1,330,201 patients with a visit in 2021. The ADI has no upper limit range, and higher values represent more deprived areas.

**Figure 2.**
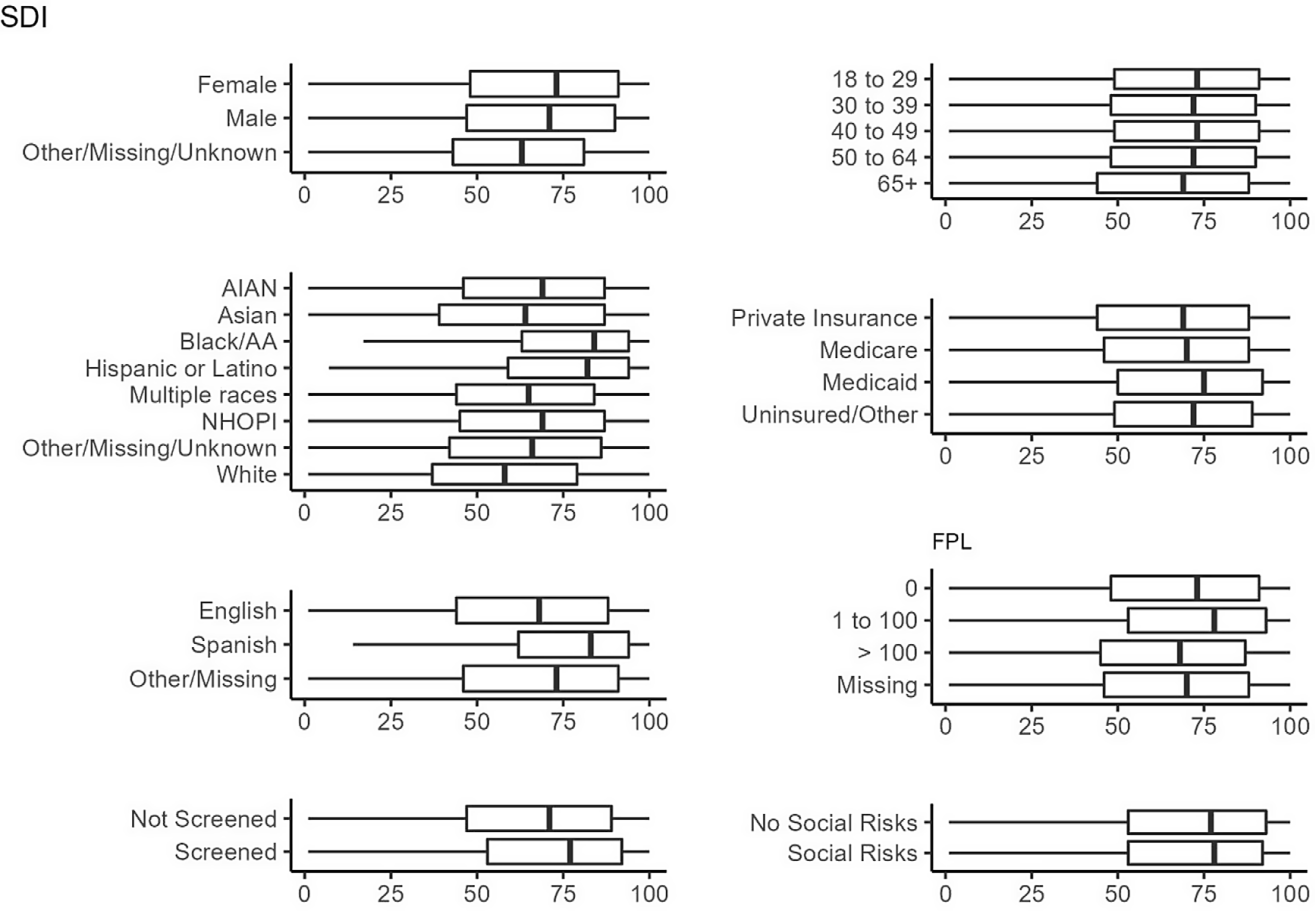
Distribution of the Social Deprivation Index (SDI) among the 1,330,201 patients with a visit in 2021. The SDI ranges from 0 to 100, where 0 indicates the least deprived and 100 indicates the most deprived area.

**Figure 3.**
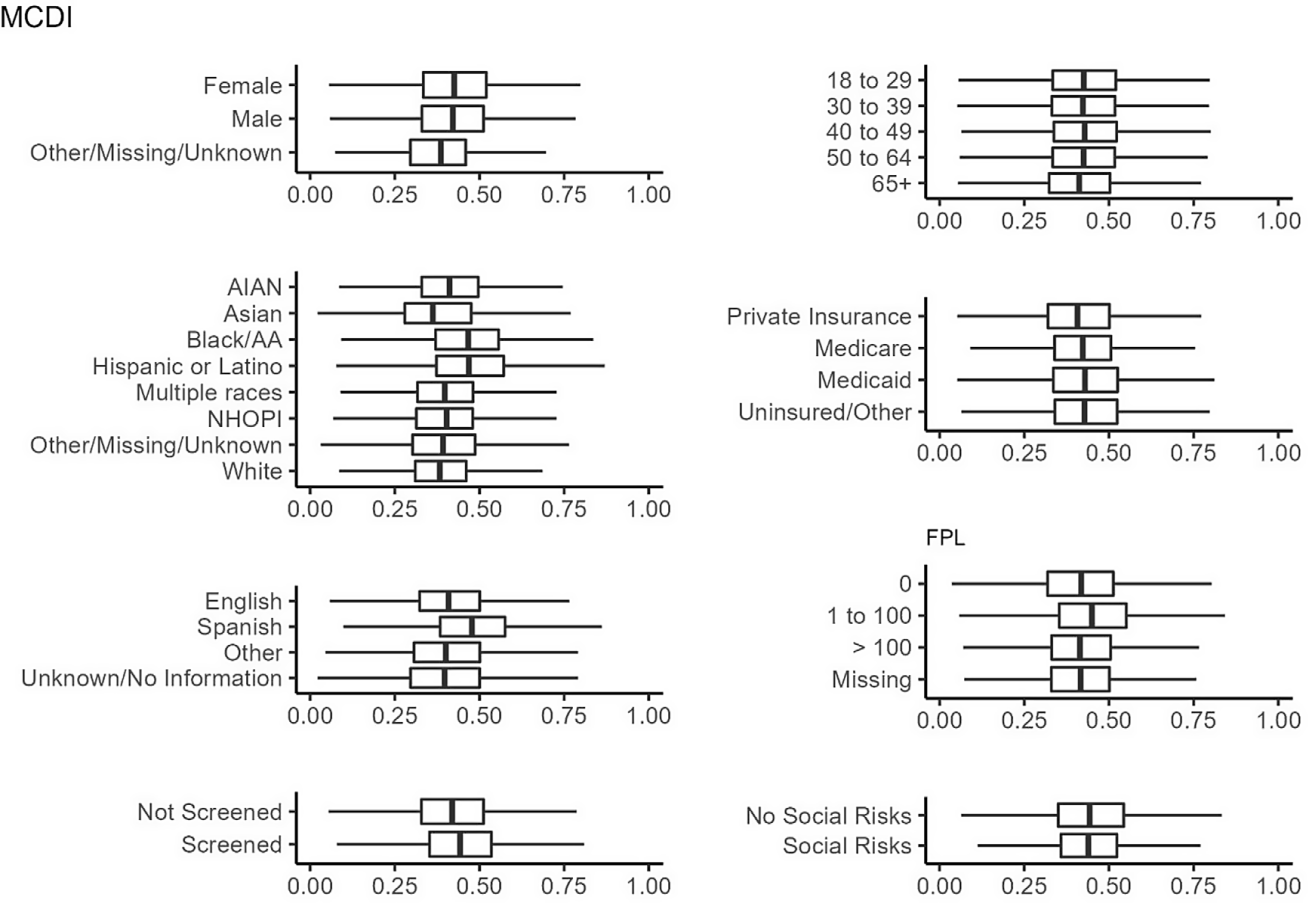
Distribution of the Material Community Deprivation Index (MCDI) among the 1,330,201 patients with a visit in 2021. The MCDI ranges from 0 to 1, where 0 indicates the least deprived and 1 indicates the most deprived area.

Figures 1–3 illustrate the distribution of the ADI, SDI, and MCDI across demographic groups, social risk screening, and social risk positivity, respectively. Variation was seen in all three measures across race/ethnicity and language categories, though more so for the ADI and SDI than the MCDI. There was little difference between the ADI, SDI, and MCDI when stratified by patients who were versus were not screened, and similarly minimal differences in the ADI, SDI, and MCDI between those with and without social risks (Figs. 1–3). Several other demographics showed little variation in the measures.

The thresholds obtained to differentiate between those with and without social risks were 134.82 (90^th^ percentile), 98 (92^nd^ percentile), and 0.56 (80^th^ percentile) for the ADI, SDI, and MCDI, respectively. Patients at or above those values were predicted to have social risks. When using only the area-level SDoH measures to predict individual social risks in our test data, low accuracy, sensitivity, and area under the curve (AUC) were observed (Table 4). The SDI had an accuracy of 0.68 and an AUC of 0.49 (Table 4), followed by the ADI with accuracy of 0.67 and an AUC of 0.47. The MCDI had the lowest performance, with an accuracy of 0.63, and an AUC of 0.48 (Table 4). There was minimal variation across the area-level SDoH measures in ability to predict individual patients’ social risks, and all three demonstrated low sensitivity (Table 4).

**Table 4.** Model accuracy, sensitivity, specificity, and area under the curve of predicting social needs from SDoH measures

	Accuracy	Sensitivity	Specificity	AUC
**ADI**	0.67 (0.66, 0.67)	0.15	0.73	0.47
**SDI**	0.68 (0.68, 0.68)	0.21	0.74	0.49
**MCDI**	0.63 (0.63, 0.63)	0.21	0.73	0.48

ADI = Area Deprivation Index; AUC = area under the curve; MCDI = Material Community Deprivation Index; SDI = Social Deprivation Index.

## Discussion

The growing national focus on measuring and addressing patients’ social risks in healthcare settings necessitates understanding the relationship between area-level SDoH measures and individual-level social risks. This study assessed this relationship in a nationwide network of community-based healthcare organizations. Its key finding is that area-level adverse SDoH are a poor proxy for individual-level social risks, consistent with recent work in this population [24] and others [25].

First, there was little difference in the distribution of area-level measures across demographic groups or among patients who were screened for individual-level risks. This demonstrates that area-level measures do not adequately identify which patients have an elevated likelihood of having social risks, as none of the three SDoH measures had predictive ability better than random chance. Rather, the measures’ low sensitivity suggests that using area-level measures would miss most individuals with social risks. This finding provides quantitative evidence of the potential for the ecological fallacy if using area-level SDoH in patient-based studies [17], as attributing area-level adverse SDoH to individual patients will substantially mischaracterize the patient’s true risk. While area-level measures remain important when measuring the community context in which an individual lives, it cannot be assumed that area-level information reflects an individual’s social needs.

There are several potential reasons why area-level measures may be a poor proxy of individual-level social risks. First, while this study measured area-level SDoH at the Census tract – a relatively small geographic level – it is possible that it still is too large of an area to finely measure SDoH. Second, often by the time these area-level measures are available for analysis, they may be outdated [37], and no longer accurately reflect that community. Finally, while social risks are influenced by the area-level SDoH they ultimately reflect the unique needs of that individual. In addition to heterogeneity across individuals, social risks may vary over time for an individual person (e.g., job loss may result in food insecurity that was not previously present). Together, these factors may contribute to the poor congruence of area-level SDoH and individual-level social risks.

These findings have implications for clinical practice, research, and policy. In clinical practice, caution should be taken when using area-level measures to identify individual patients with social risks. Healthcare systems seeking to understand their patients’ social risks [38,39] may wish to use area-level SDoH data as they are readily available, but these results suggest that area-level adverse SDoH do not always predict individual-level social risks. When area-level measures are used to adjust for patient social complexity in research, this adjustment applies only to the *area* in which someone lives, not to individual-level social risks. Conceptually, this may be appropriate at times, but *area* and *individual* measurement and interpretation are not interchangeable. Researchers should consider further examining how using individual-level measures, in addition to and in lieu of area-level measures, changes result interpretations. These findings also suggest a need for caution when using area-level SDoH indices in policy, quality measures, and payment structures [9,10,40–43].

In addition, an association was seen between demographic factors (i.e., sex, race/ethnicity, language, age, payer) and likelihood of being screened for social risks and reporting social risks, consistent with previous analyses in the same health center network [44]. Results add to prior work by identifying potential differences in which patients are screened for social risks as well as high between-health system variation in screening. This illuminates the need to consider sources of screening biases *within* health centers, as individual-level screening may not be occurring equitably and suggests that reducing between-health center differences may require explicating how varying health center practices impact screening patterns. In addition to health center screening practices contributing to these differences, patient comfort and potential stigma may influence efforts to routinize social risk screening. Future research examining screening and positivity in multi-institutional datasets should consider assessing contextual data on screening practices and other influential health center-level factors.

Some demographic factors (i.e., males, uninsured/other payer, at or below the FPL) associated with lower odds of screening were also associated with higher odds of being positive for social risks, while others (i.e., language) were associated with higher odds of screening and lower odds of social risk positivity. This both underscores concerns about the equitable nature of social risk screening and highlights the potential influence of screening equity on population estimates of social risk prevalence. Taken together with the findings that using *area-level* measures is likely to obscure individual patients’ needs, policymakers, and payment administrators should incentivize improving routine *individual-level* social risk data collection and reporting. For example, two recently released quality measures are available for a few payment programs that specifically focus on social risk screening and positivity [12,13]. Concurrently, we must support those care settings that serve populations with a high prevalence of these social risks in collecting these data [45].

A study strength is our use of data from a nationwide network of community-based healthcare organizations that have been innovators in social risk screening since 2016 [22,46,47]. Studies indicate that community-based health centers have higher rates of social risk screening than other care settings, although screening behaviors and capacity vary widely [48]. As these organizations operate independently, despite sharing a single instance of Epic, we were able to assess health system-level variation. A study limitation is that all organizations in this study were community-based healthcare organizations; findings may not apply to other settings, systems, and patients. Further, we only included health systems that had at least one social risk screening, excluding organizations that never screen. Understanding why some organizations do not screen may help illuminate barriers to routine screening. Furthermore, some variables (e.g., FPL) which are collected during clinical visits have varied completeness. This is an inherent limitation of working with EHR data and should continue to be addressed. Despite these limitations, this study provides important quantitative findings that should reinforce the caution needed when using area-level SDoH data in health care.

## Conclusion

Three different and commonly used area-level measures of adverse SDoH are poor proxies for individual-level social risk factors. While area-level SDoH measures provide valuable information about the communities where patients live, caution should be taken when using area-level adverse SDoH measures to assume individual-level social risks.

## Supporting information

Bensken et al. supplementary material Bensken et al. supplementary material

## Supplementary material

The supplementary material for this article can be found at https://doi.org/10.1017/cts.2023.680.
