# Supplementary material for: Area-level social determinants of health and individual-level social risks: Assessing predictive ability and biases in social risk screening
Source: J Clin Transl Sci. 2023 Nov 10;7(1):e257. doi: 10.1017/cts.2023.680 (PMC10790234; doi:10.1017/cts.2023.680)

Supplemental Table 1. Overlap of race/ethnicity and preferred language.

|  | English | Spanish | Other | Unknown/No Information |
| --- | --- | --- | --- | --- |
| n (column %) | 922,028 | 304,612 | 91,900 | 11,661 |
| Race/Ethnicity |  |  |  |  |
| AIAN | 6,628 (0.7) | 108 (0.04) | 228 (0.2) | 54 (0.5) |
| Asian | 43,508 (4.7) | 150 (0.03) | 50,559 (55.0) | 636 (5.5) |
| Black/AA | 199,058 (21.6) | 588 (0.2) | 19,320 (21.0) | 1,625 (13.9) |
| Hispanic or Latino | 165,814 (18.0) | 283,271 (93.0) | 2,297 (2.5) | 1,566 (13.4) |
| Multiple races | 10,562 (1.1) | 49 (0.02) | 364 (0.4) | 79 (0.7) |
| NHOPI | 3,886 (0.4) | 79 (0.03) | 682 (0.7) | 40 (0.3) |
| Other/Missing/Unknown | 69,252 (7.5) | 10,908 (3.6) | 6,935 (7.5) | 3,935 (33.7) |
| White | 423,320 (45.9) | 9,459 (3.1) | 11,515 (12.5) | 3,726 (32.0) |

AIAN: American Indian or Alaska Native; AA: African American; NHOPI: Native Hawaiian or Other Pacific Islander.

Supplemental Table 2. Comparison of Training and Test Samples

|  | **Training** | **Test** |
| --- | --- | --- |
|  | 170,908 | 73,247 |
| **Sex, n (%)** |  |  |
| Female | 114079 (66.7) | 48697 (66.5) |
| Male | 56714 (33.2) | 24490 (33.4) |
| Other/Missing/Unknown | 115 (0.1) | 60 (0.1) |
| **Race/Ethnicity, n (%)** |  |  |
| AIAN | 785 (0.5) | 367 (0.5) |
| Asian | 7282 (4.3) | 3133 (4.3) |
| Black/AA | 33518 (19.6) | 14324 (19.6) |
| Hispanic or Latino | 62765 (36.7) | 26792 (36.6) |
| Multiple races | 1233 (0.7) | 530 (0.7) |
| NHOPI | 439 (0.3) | 211 (0.3) |
| Other/Missing/Unknown | 8310 (4.9) | 3653 (5.0) |
| White | 56576 (33.1) | 24237 (33.1) |
| **Age, n (%)** |  |  |
| 18 to 29 | 36182 (21.2) | 15536 (21.2) |
| 30 to 39 | 35362 (20.7) | 15256 (20.8) |
| 40 to 49 | 34842 (20.4) | 14818 (20.2) |
| 50 to 64 | 41390 (24.2) | 17711 (24.2) |
| 65+ | 23132 (13.5) | 9926 (13.6) |
| **Language, n (%)** |  |  |
| English | 118301 (69.2) | 50709 (69.2) |
| Spanish | 42397 (24.8) | 18121 (24.7) |
| Other | 9294 (5.4) | 4018 (5.5) |
| Unknown/No Information | 916 (0.5) | 399 (0.5) |
| **FPL Percentage** |  |  |
| Mean (Std Dev) | 107.90 (317.98) | 108.93 (322.70) |
| Median [IQR] | 60.00 [0.00, 118.00] | 61.00 [0.00, 118.00] |
| 0 | 41772 (24.4) | 17974 (24.5) |
| 1 to 100 | 46695 (27.3) | 19620 (26.8) |
| > 100 | 39341 (23.0) | 17009 (23.2) |
| Missing | 43100 (25.2) | 18644 (25.5) |
| **Payer, n (%)** |  |  |
| Private Insurance | 40828 (23.9) | 17633 (24.1) |
| Medicare | 22737 (13.3) | 9677 (13.2) |
| Medicaid | 76392 (44.7) | 32830 (44.8) |
| Uninsured/Other | 30951 (18.1) | 13107 (17.9) |
| **Social Risk** |  |  |
| Positive | 42744 (25.0) | 18670 (25.5) |
| **Area Deprivation Index (ADI)** |  |  |
| Mean (Std Dev) | 109.19 (20.30) | 109.14 (20.34) |
| Median [IQR] | 108.79 [95.15, 122.30] | 108.74 [94.84, 122.24] |
| **Social Deprivation Index (SDI)** |  |  |
| Mean (Std Dev) | 70.16 (25.94) | 70.17 (25.93) |
| Median [IQR] | 77.00 [53.00, 92.00] | 78.00 [53.00, 92.00] |
| **Material Community Deprivation Index (MCDI)** |  |  |
| Mean (Std Dev) | 0.45 (0.13) | 0.45 (0.13) |
| Median [IQR] | 0.44 [0.35, 0.54] | 0.44 [0.35, 0.53] |

AIAN: American Indian or Alaska Native; AA: African American; NHOPI: Native Hawaiian or Other Pacific Islander.

Supplemental Table 3. Full models including language instead of race/ethnicity.

|  | **Screened for Social Risks** | **Positive for Social Risk** |
| --- | --- | --- |
|  | ICC = 0.55 | ICC = 0.38 |
| **Sex** |  |  |
| Female | *Ref* | *Ref* |
| Male | 0.58 (0.58, 0.59) | 1.23 (1.21, 1.26) |
| Other/Missing | 1.20 (1.01, 1.43) | 1.91 (1.36, 2.68) |
| **Language** |  |  |
| English | *Ref* | *Ref* |
| Spanish | 1.12 (1.11, 1.14) | 0.84 (0.82, 0.87) |
| Other | 1.11 (1.09, 1.14) | 0.61 (0.58, 0.64) |
| Unknown/No Information | 0.64 (0.60, 0.68) | 1.01 (0.87, 1.17) |
| **Age** |  |  |
| 18 to 29 | *Ref* | *Ref* |
| 30 to 39 | 1.12 (1.11, 1.14) | 1.11 (1.07, 1.15) |
| 40 to 49 | 1.28 (1.26, 1.30) | 1.22 (1.18, 1.26) |
| 50 to 64 | 0.89 (0.88, 0.90) | 1.57 (1.52, 1.62) |
| 65+ | 0.85 (0.83, 0.86) | 0.93 (0.89, 0.97) |
| **Payer** |  |  |
| Private Insurance | *Ref* | *Ref* |
| Medicare | 1.37 (1.34, 1.40) | 1.49 (1.42, 1.55) |
| Medicaid | 1.24 (1.22, 1.25) | 1.90 (1.85, 1.96) |
| Uninsured/Other | 0.83 (0.81, 0.84) | 1.90 (1.83, 1.97) |
| **FPL Percentage** |  |  |
| > 100 | *Ref* | *Ref* |
| 0 | 0.85 (0.83, 0.86) | 1.42 (1.37, 1.47) |
| 1 to 100 | 0.97 (0.95, 0.98) | 1.35 (1.31, 1.40) |
| Missing | 0.75 (0.73, 0.76) | 1.26 (1.22, 1.30) |

Supplemental Table 4. Distribution of ADI, SDI, and MCDI by Demographics and Social Risks Screening and Positivity

|  | **Area Deprivation Index (ADI)** | | **Social Deprivation Index (SDI)** | | **Material Community Deprivation Index (MCDI)** | |
| --- | --- | --- | --- | --- | --- | --- |
|  | Mean (Std Dev) | Median [IQR] | Mean (Std Dev) | Median [IQR] | Mean (Std Dev) | Median [IQR] |
| **Sex** |  |  |  |  |  |  |
| Female | 105.6 (20.7) | 104.3 [91.2, 119.0] | 67.2 (26.2) | 73.0 [48.0, 91.0] | 0.43 (0.13) | 0.43 [0.33, 0.52] |
| Male | 104.7 (20.6) | 103.2 [90.7, 117.7] | 66.3 (26.3) | 71.0 [47.0, 90.0] | 0.43 (0.13) | 0.42 [0.33, 0.51] |
| Other/Missing | 96.0 (17.0) | 95.1 [84.5, 106.3] | 59.9 (24.4) | 63.0 [43.0, 81.0] | 0.39 (0.11) | 0.39 [0.30, 0.46] |
| **Race/Ethnicity** |  |  |  |  |  |  |
| AIAN | 102.2 (18.6) | 101.4 [90.4, 113.9] | 64.4 (25.6) | 69.0 [46.0, 87.0] | 0.42 (0.12) | 0.41 [0.33, 0.50] |
| Asian | 95.6 (20.7) | 95.1 [80.7, 110.9] | 60.8 (27.8) | 64.0 [39.0, 87.0] | 0.37 (0.13) | 0.36 [0.28, 0.48] |
| Black/AA | 112.5 (20.0) | 113.4 [98.0, 125.2] | 76.2 (23.2) | 84.0 [63.0, 94.0] | 0.47 (0.13) | 0.47 [0.37, 0.56] |
| Hispanic or Latino | 111.2 (21.3) | 110.7 [96.0, 125.2] | 74.3 (23.6) | 82.0 [59.0, 94.0] | 0.47 (0.14) | 0.47 [0.37, 0.57] |
| Multiple races | 100.0 (18.3) | 99.1 [88.1, 111.2] | 62.3 (25.3) | 65.0 [44.0, 84.0] | 0.40 (0.12) | 0.40 [0.32, 0.48] |
| NHOPI | 99.8 (18.3) | 99.5 [88.0, 111.4] | 63.9 (25.8) | 69.0 [45.0, 87.0] | 0.40 (0.12) | 0.40 [0.31, 0.48] |
| Other/Missing/Unknown | 99.6 (19.8) | 98.7 [85.6, 112.9] | 62.2 (26.8) | 66.0 [42.0, 86.0] | 0.40 (0.13) | 0.39 [0.30, 0.49] |
| White | 99.0 (17.2) | 98.5 [88.1, 109.6] | 57.0 (25.9) | 58.0 [37.0, 79.0] | 0.39 (0.11) | 0.38 [0.31, 0.46] |
| **Age** |  |  |  |  |  |  |
| 18 to 29 | 105.7 (20.8) | 104.4 [91.2, 119.0] | 67.6 (26.0) | 73.0 [49.0, 91.0] | 0.43 (0.13) | 0.43 [0.33, 0.52] |
| 30 to 39 | 105.4 (20.6) | 104.0 [91.0, 118.8] | 67.2 (26.0) | 72.0 [48.0, 90.0] | 0.43 (0.13) | 0.42 [0.33, 0.52] |
| 40 to 49 | 105.9 (20.5) | 104.7 [91.5, 119.0] | 67.8 (25.9) | 73.0 [49.0, 91.0] | 0.44 (0.13) | 0.43 [0.34, 0.52] |
| 50 to 64 | 105.3 (20.7) | 104.1 [91.2, 118.5] | 66.8 (26.3) | 72.0 [48.0, 90.0] | 0.43 (0.13) | 0.42 [0.33, 0.52] |
| 65+ | 103.1 (20.6) | 101.9 [89.7, 116.6] | 63.9 (27.1) | 69.0 [44.0, 88.0] | 0.42 (0.13) | 0.41 [0.32, 0.50] |
| **Language** |  |  |  |  |  |  |
| English | 103.4 (20.2) | 101.9 [89.8, 116.7] | 64.0 (26.6) | 68.0 [44.0, 88.0] | 0.42 (0.13) | 0.41 [0.32, 0.50] |
| Spanish | 111.9 (20.9) | 111.6 [97.5, 125.3] | 75.6 (22.8) | 83.0 [62.0, 94.0] | 0.48 (0.13) | 0.48 [0.38, 0.58] |
| Other | 101.2 (19.9) | 100.9 [87.7, 115.7] | 67.0 (26.9) | 74.0 [48.0, 91.0] | 0.40 (0.13) | 0.40 [0.31, 0.50] |
| Unknown/No Information | 100.6 (21.0) | 99.6 [85.4, 114.3] | 61.6 (28.0) | 64.0 [39.0, 88.0] | 0.40 (0.13) | 0.40 [0.30, 0.50] |
| **Payer** |  |  |  |  |  |  |
| Private Insurance | 103.3 (19.9) | 102.1 [89.7, 116.6] | 64.1 (26.8) | 69.0 [44.0, 88.0] | 0.42 (0.13) | 0.41 [0.32, 0.50] |
| Medicare | 105.1 (19.6) | 103.6 [91.9, 117.4] | 65.4 (26.2) | 70.0 [46.0, 88.0] | 0.43 (0.13) | 0.42 [0.34, 0.51] |
| Medicaid | 106.1 (21.6) | 104.7 [91.1, 120.5] | 68.7 (26.1) | 75.0 [50.0, 92.0] | 0.44 (0.14) | 0.43 [0.34, 0.53] |
| Uninsured/Other | 105.5 (20.1) | 104.6 [91.7, 118.3] | 66.9 (25.6) | 72.0 [49.0, 89.0] | 0.44 (0.13) | 0.43 [0.34, 0.52] |
| **FPL Percentage** |  |  |  |  |  |  |
| 0 | 103.7 (21.9) | 102.6 [88.0, 118.3] | 67.1 (26.6) | 73.0 [48.0, 91.0] | 0.42 (0.14) | 0.42 [0.32, 0.51] |
| 1 to 100 | 108.9 (21.1) | 108.4 [94.1, 122.8] | 70.8 (25.5) | 78.0 [53.0, 93.0] | 0.46 (0.14) | 0.45 [0.35, 0.55] |
| > 100 | 103.6 (19.4) | 102.1 [90.7, 115.8] | 64.1 (26.1) | 68.0 [45.0, 87.0] | 0.42 (0.13) | 0.41 [0.33, 0.50] |
| Missing | 104.4 (19.5) | 103.0 [91.2, 117.0] | 64.9 (26.4) | 70.0 [46.0, 88.0] | 0.42 (0.13) | 0.42 [0.33, 0.50] |
| **Social Risk, Screened** |  |  |  |  |  |  |
| No | 104.3 (20.7) | 102.9 [90.3, 117.6] | 66.1 (26.3) | 71.0 [47.0, 89.0] | 0.43 (0.13) | 0.42 [0.33, 0.51] |
| Yes | 109.2 (20.3) | 108.8 [95.1, 122.3] | 70.2 (25.9) | 77.0 [53.0, 92.0] | 0.45 (0.13) | 0.44 [0.35, 0.54] |
| **Social Risk, Positive** |  |  |  |  |  |  |
| No | 109.5 (21.0) | 108.9 [94.8, 122.9] | 70.1 (26.2) | 77.0 [53.0, 93.0] | 0.45 (0.14) | 0.44 [0.35, 0.54] |
| Yes | 108.1 (18.1) | 108.5 [95.4, 120.2] | 70.4 (25.2) | 78.0 [53.0, 92.0] | 0.44 (0.12) | 0.44 [0.36, 0.52] |

Std Dev: Standard Deviation; IQR: Interquartile Range; AIAN: American Indian or Alaska Native; AA: African American; NHOPI: Native Hawaiian or Other Pacific Islander.

Supplemental Figure 1. Example of Study Timeline. We took each patient’s latest visit in 2021, then did a 12-month lookback period for all social risk screening in that time frame. For example, Patient A has a visit in March of 2021 and all social risk screenings were identified for this patient from March 2020 – March 2021. Similarly, Patient B had a visit in November of 2021 and their lookback period was November 2020 to November 2021.


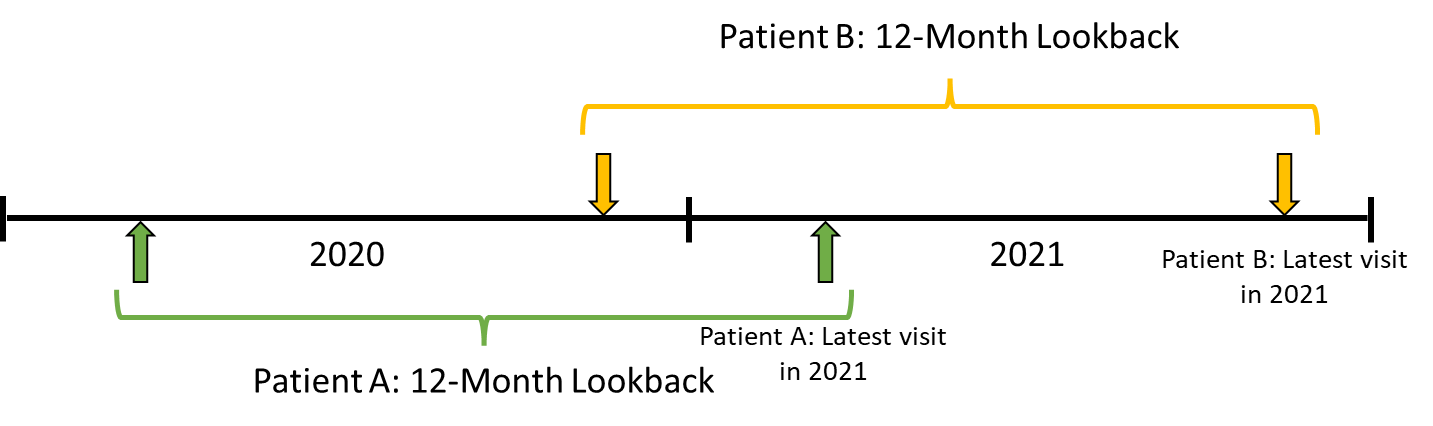

Supplement: Bensken et al. supplementary material [file S2059866123006805sup001.docx]
